# Ribosomal Intergenic Spacer Analysis as a Tool for Monitoring Methanogenic *Archaea* Changes in an Anaerobic Digester

**DOI:** 10.1007/s00284-013-0353-2

**Published:** 2013-03-23

**Authors:** Slawomir Ciesielski, Katarzyna Bułkowska, Dorota Dabrowska, Dariusz Kaczmarczyk, Przemyslaw Kowal, Justyna Możejko

**Affiliations:** 1 Department of Environmental Biotechnology, Faculty of Environmental Sciences, University of Warmia and Mazury in Olsztyn, Olsztyn, Poland; 2 Department of Microbiology, Faculty of Biology and Biotechnology, University of Warmia and Mazury in Olsztyn, Olsztyn, Poland

## Abstract

The applicability of a newly-designed PCR primer pair in examination of methanogenic *Archaea* in a digester treating plant biomass was evaluated by Ribosmal Intergenic Spacer Analysis (RISA). To find a suitable approach, three variants of RISA were tested: (1) standard, polyacrylamide gel-based, (2) automated, utilized capillary electrophoresis (GA-ARISA), and (3) automated microfluidics-based (MF-ARISA). All three techniques yielded a consistent picture of archaeal community structure changes during anaerobic digestion monitored for more than 6 weeks. While automated variants were more practical for handling and rapid analysis of methanogenic *Archaea*, the gel-based technique was advantageous when micro-organism identification was required. A DNA-sequence analysis of dominant bands extracted from the gel revealed that the main role in methane synthesis was played by micro-organisms affiliated with *Methanosarcina barkeri*. The obtained results revealed that RISA is a robust method allowing for detailed analysis of archaeal community structure during organic biomass conversion into biogas. In addition, our results showed that GA-ARISA has a higher resolution and reproducibility than other variants of RISA and could be used as a technique for tracking changes in methanogenic *Archaea* in an anaerobic digester.

## Introduction

Successful anaerobic treatment of organic wastes requires the stable functioning of a complex, interdependent microbial community [7, 8]. The degradation of the organic compounds to carbon dioxide and methane occurs in four, discrete steps that are carried out by different groups of micro-organisms. At the beginning, organic molecules such as complex carbohydrates, proteins and lipids are hydrolyzed into their components [17]. The generated monomers and oligomers, such as amino acids, simple carbohydrates, and fatty acids are converted into organic alcohols, volatile fatty acids, hydrogen, and carbon dioxide. Next, the digested products are further degraded into acetate, hydrogen, and carbon dioxide. The final step is methanogenesis, which results in the production of methane and carbon dioxide from either acetate or hydrogen/formate and carbon dioxide [9, 19]. This step is carried out by methanogens, which are especially important because methanogenesis is often the rate-limiting step in anaerobic treatment of wastes [7]. Methanogenic micro-organisms belong to *Archaea*, a unique prokaryotic domain of life. This group contains: (i) the acetotrophic methanogens, (ii) hydrogenotrophic methanogens, and (iii) methylotrophs which convert methyl compounds such as methanol and methylamines. Methane-producing micro-organisms are obligate anaerobes and are very sensitive to environmental changes [14]. Because methanogenesis is usually the rate-limiting step in the overall process, the appropriate control of the methanogenic phase has been a key factor in the successful operation of anaerobic processes [20]. Therefore, understanding the behavior of the archaeal community is crucial to optimize the anaerobic process for biogas production.

Recently, a study of complex microbial communities has been performed by the application of culture-independent, molecular methods based on directed analysis of the 16S rRNA gene structure. Among them, Denaturing Gradient Gel Electrophoresis (DGGE) analysis is one of the most widely-used molecular techniques, enabling the identification of community members by the recovery and sequencing of amplification products. This genetic fingerprinting approach is useful for comparisons of microbial communities from different environments or in following changes in community structures over time [10]. However, DGGE has many limitations, such as limited sensitivity of detection for some rare community members and the co-migration of DNA fragments with different sequences. Thus, other DNA fingerprinting techniques have been tested for application in microbial community analysis. An alternative technique is Ribosomal Intergenic Spacer Analysis (RISA), which is based on the amplification of the intergenic region located between the *16S* and *23S rRNA* genes in the rRNA operon. This region is characterized by significant variability in the length and nucleotide sequence among different microbial genotypes [5]. Recently, the separation process of amplified DNA has been improved by involving a fluorescence-tagged oligonucleotide primer for PCR amplification and subsequent electrophoresis in an automated system. Due to the high resolution of the gels and the high sensitivity of the fluorescence detection, numbers of founded peaks are much higher in Automated Ribosomal Intergenic Spacer Analysis (ARISA) than in RISA profiles [12].

The main goal of this study was to develop a method facilitating the examination of changes in the methanogenic Archaea community during anaerobic digestion by RISA. The possibility of applying both traditional RISA and its automated versions (ARISA) was explored.

## Materials and Methods

### Anaerobic Digester Set-up

Biomass for the anaerobic digestion were silages of *Zea mays* L. mixed with alfalfa (*Medicago sativa*) cultivar Legend in the ratio of 9:1 by wVS/wVS. *Medicago sativa*, before ensiling was mixed with timothy grass (*Phleum pratense*) cultivar Climax. The plant biomass was obtained from field experiments performed in 2010 in the Production and Experimental Station at Bałcyny (53°35′49′′N, 19°51′20.3′′E), University of Warmia and Mazury in Olsztyn (Poland). The crops were harvested by self-propelled harvesters equipped with cutting drums that chopped the crops into pieces 2–3 cm in length. Next, the raw, harvested crops were ensiled by concentrating in 200 L silos lined with foil for 90 day. Formic acid (85 %) was added at a ratio of 5 g acid to 1 kg biomass. For feedstock standardization, the silages were dried at 60 °C and ground in a cutting mill (Retsch SM100, Germany), passed through a 1 mm screen and stored in plastic containers at room temperature. As co-substrates, the pig manure and spent wash, obtained from the agriculture farm in Gwiździny (Warmia-Masuria Province, North-East Poland) were used. The pig manure and spent wash content (as % of volatile solids) in the digester were 6.5 % each.

**Fig. 1:**
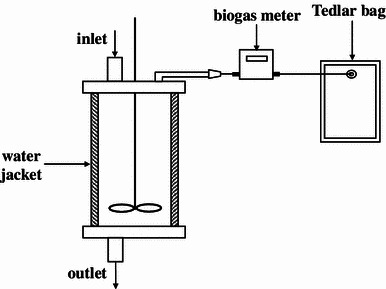
Scheme of CSTR reactor

Anaerobic digestion were evaluated in anaerobic, continuously stirred tank reactor (CSTR) with working volume of 6 L (Fig. 1), operated in semi-continues mode at 39 °C, constant hydraulic retention time (45 day) and organic loading rate (2.02 g/L day). Reactor was inoculated with anaerobic sludge from the sludge digestion chambers of a municipal wastewater treatment plant in Olsztyn (North-East Poland). Once a day reactor was supplied with 133.3 mL of the feedstock after the same volume of digestate had been withdrawn. The chemical–physical characterization of the feedstock was shown in Table 1.

**Table 1:** Chemical–physical characterization of feedstock (standard deviations for triplicates are shown in parentheses)

Parameters	Unit	Maize + Alfalfa + Spent wash + Pig manure
pH	–	5.64 (± 0.07)
Alkalinity	mval/L	65.75 (± 4.79)
	mg CaCO_3_/L	3287.5 (± 239)
Volatile fatty acids (VFAs)	mg/L	3,034 (± 202.8)
COD	mg O_2_/L	31,471 (± 2,929)
Ammonia	mg N–NH_4_^+^/L	576.8 (± 40.45)
TS	g/kg	99.73 (± 4.56)
VS	g/kg	90.98 (± 4.14)

Parameters recorded in digestate at the time of sampling were shown in Table 2. Analytical process control involved analysis of total solids (TS), volatile solids (VS), chemical oxygen demand (COD), volatile fatty acids (VFAs), and N–NH_4_^+^, that were determined according to standard methods [2]. The pH was measured immediately after sampling using a pH meter (Hanna HI 221, USA). The total alkalinity was measured by titration to pH 4.3 with normalized 0.1 M HCl using a Schott titroline system. Analyses for COD, VFAs, N–NH_4_^+^, and alkalinity were performed for filtered supernatant samples previously centrifuged at 8,693×*g* for 10 min. The biogas volume was measured using gas meter (Aalborg, model XFM 17). Methane and carbon dioxide content were measured for samples collected in Tedlar bags by automatic analyzer GA2000+ (Geotechnic Instruments).

**Table 2:** Process parameters recorded at the time of sampling

Parameters	Unit	Inoculum	1st day	10th day	22nd day	30th day	43rd day
TS	g/L	28.30	36.20	30.85	29.95	31.00	35.55
VS	g/L	23.30	26.10	21.85	22.65	24.20	27.85
pH	–	7.28	7.38	7.51	7.55	7.60	7.60
COD	mg O_2_/L	2,479	2,000	3,033	2,899	2,941	2,815
VFAs	mg/L	1,611	1,543	1,371	1,423	1,611	1,457
Ammonia	mg N–NH_4_^+^/L	389	417	504	661	812	770
Biogas production	L/day	–	0.15	8.45	8.22	7.67	8.23
Methane production	L/day	–	0.08	5.23	5.16	4.84	5.27

### DNA Extraction

Approximately 1 mL aliquots of well-homogenized sludge were immediately frozen at the time of sampling and stored at −20 °C. Extraction of total DNA was performed as follows: 0.075 g of biomass sample was washed in sodium phosphate buffer (0.1 M; pH 8.0) and pelleted by centrifugation. After rejection of the supernatant 1 mL of the extraction buffer (100 mM Tris–HCl; 100 mM EDTA; 1.5 M NaCl; pH = 8) and 0.3 g of glass beads (Ø 0.25–0.5; Carl Roth, Germany) were added. Samples were shaken for 20 min. at 5,000 rpm in bead beating device (Uniequip, Germany). To improve the process of cell disruption, samples were additionally incubated for 1 h in 65 °C in the presence of 0.2 mL of the 10 % sodium dodecyl sulfate (SDS) solution. In the following step proteins and other impurities were pelleted by centrifugation (10 min, 13,000 rpm) and DNA solution was purified in silica washing columns (A&A Biotechnology, Poland). After purification DNA was suspended in 100 μL of sterile, DNAase free water and stored in −20 °C.

### Primer Design

Specific primers spanning Ribosomal Intergenic Spacer region were designed on the base of DNA sequences available in the Genbank (National Center for Biotechnology Information, NCBI). The following DNA sequences were studied for the design of primer specific for *16S rRNA* gene: *Methanobacterium* sp. OM15 (Acc. Nr AJ550160), *Methanothermobacter* sp. RY3 (Acc. Nr FJ418154), *Methanococcus voltae* (Acc. Nr MVU38488, uncultured *Methanosarcina* sp. clone TS1A083 (Acc. Nr JF789590), *Methanosaeta harundinacea* strain 6Ac (Acc. Nr AY970347), uncultured *Methanosphaera* sp. clone 24 (Acc. Nr DQ402032). For primer recognizing 23S rRNA gene following DNA sequences were used: *Methanobacterium* sp. SWAN-1 (Acc. Nr CP002772), *Methanosphaera stadtmanae* DSM 3091 (Acc. Nr CP000102), *Methanococcus maripaludis* X1 (Acc. Nr CP002913), *Methanotorris igneus* Kol 5 (Acc. Nr CP002737), *Methanosarcina barkeri* str. Fusaro (Acc. Nr CP000099), *Methanosaeta concilii* GP-6 (Acc. Nr CP002565), *Methanopyrus kandleri* AV19 (Acc. Nr AE009439), *Methanothermobacter thermautotrophicus* str. Delta H (Acc. Nr AE000666), uncultured archaeon fos0625e3 (Acc. Nr CR937011). From the corresponding consensus sequences, several regions were selected for further primer design (Table 3). Selected regions were subjected to similarity searches using the BLASTN algorithm (NCBI) to check possible matches with sequences from other organisms. The probabilities of primer-dimer formation and autofolding were also studied to keep them as low as possible. Primers designed and selected for this study were: 16S-RIS-M (5′-TGA AGC TGG AAT (CG)C GTA GTA ATC GC-3′), and 23S-RIS-M (5′-CTA AGA TGT TTC AAT(CT)C(CGA) (CG)(AGTC)(AG) (CG)GT TCC-3′). Primers were synthesized by GENOMED (Poland).

**Table 3:**
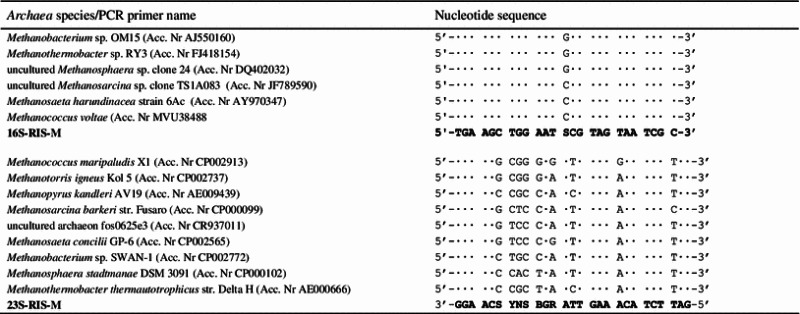
Alignment of *Archaea* nucleotide sequences that were used to design the 16S-RIS-M and 23-RIS-M PCR primers

Next to the latin names are indicated the GenBank accession numbers. Dots indicate identity of rDNA and primers sequences

S (C or G), Y (C or T), R (G or A), V (C or G or A), B (C or G or T), N (C or G or T or A)

### Polymerase Chain Reaction

Ribosomal Intergenic Spacer was amplified using designed primers pair (16S-RIS-M and 23-RIS-M). PCR was performed in Eppendorf^®^ Mastercycler Gradient (Eppendorf, Germany). The mixtures used for PCR amplification contained 50 ng of extracted total DNA, 0.5 μM of each primer, 150 μM of deoxynucleoside triphosphate (Promega, USA), 1.5 U of GoTaq Flexi DNA polymerase (Promega, USA), 5 μL of GoTaq Flexi colorless reaction buffer (500 mM KCl, pH 8.5; Triton X-100), 1.5 mM MgCl_2_, and sterile water to a final volume of 30 μL. The temperature program for DNA amplification was as follows: 94 °C for 5 min; 35 cycles of denaturation at 94 °C for 45 s, annealing at 50 °C for 1 min, extension at 72 °C for 1 min and a single final elongation at 72 °C for 10 min. The PCR amplicons of RIS were checked by resolving on 1.5 % agarose gels stained with ethidium bromide.

### Ribosomal Intergenic Spacer Analysis

After successful DNA amplification, the PCR products were separated in 8 % polyacrylamide gel (29:1 acrylamide:bisacrylamide). Electrophoresis was carried out at 80 V for 180 min in 1× TBE buffer (89 mM Tris base, 89 mM Boric acid, 2 mM EDTA; pH 8.0). The size of PCR products was estimated using a molecular weight marker (100 bp Ladder; Promega, USA). Stained with SybrGold (Invitrogen), gel was viewed with an ultraviolet transiluminator and recorded with a CCD camera (Gel Logic 200, Eastman Kodak Company, USA). Bands were detected automatically from the digital images of the gel using KODAK 1D 3.6 Image Analysis Software (Eastman Kodak Company, USA).

### Microfluidics-based Automated Ribosomal Intergenic Spacer Analysis (MF-ARISA)

PCR was performed in 30 μL volumes under the same conditions described above. Each reaction mixture was loaded into chip wells that were prepared according to the manufacturer’s recommendations (DNA 1000 LabChip kit; Agilent Technologies, USA). Samples were analyzed using an Agilent 2100 Bioanalyzer and the Agilent 2100 Expert software program. The Agilent software determined peak sizes and areas based on data for internal size standards in each lane (15 and 1,500 bp) and an external ladder. To include the maximum number of peaks while excluding background fluorescence, a threshold of 20 fluorescence units greater than the baseline was set manually. Peak sizes were compared for all samples; the values within ±5 % were assumed to be the same, according to the kit instructions. The relative amount of each DNA fragment in the PCR amplicons was estimated as the ratio between the fluorescence (peak area) of the DNA fragment of interest and the total fluorescence of all DNA fragments in the profile.

Genetic Analyzer-based Automated Ribosomal Intergenic Spacer Analysis (GA-ARISA) was based on the method described by Fisher and Triplett [5]. Primer 16S-RIS-M was 5′ labeled with the fluorochrome 6FAM (GENOMED, Poland). PCR was performed in 30 μL volumes under the same conditions described above. 1 μL of amplicons was mixed with 0.5 μL of GeneScan 1200LIZ size standard (Applied Biosystems, USA) and 13.5 μL Hi-Di formamide (Applied Biosystems, USA), then denatured at 94 °C for 5 min, and chilled on ice for 5 min. Sample fragments were discriminated using the Applied Biosystems 3130 Genetic Analyzer (Applied Biosystems, USA) and Genetic Analyzer Data Collection 3.0 software (Applied Biosystems, USA). The length of the PCR fragments was analyzed using Applied Biosystems 3130 Genetic Analyzer (Applied Biosystems) in AFLP mode. The separation was performed in 36-cm capillaries array, and POP-7 polymer (Applied Biosystems). The length of injection time was 42 s, and 15 kV injection voltage, 15 kV run voltage was applied. Amplicon sizes between 100 and 1,000 bp were determined using GeneMapper 4.0 analytical software (Applied Biosystems). To include the maximum number of peaks while excluding background fluorescence, only fragments above a threshold of 200 fluorescence units were taken under consideration. Similarly to Agilent Technologies approach, the relative amount of each fragment in the PCR product was estimated as the ratio between the fluorescence (peak area) of the fragment of interest and the total fluorescence of all fragments in the profile.

### Analysis of Banding Profiles

Changes in microbial community structure during anaerobic digestion was analyzed by calculating genetic distance between samples. Using the presence–absence data the banding patterns were converted to a binary matrix, which allowed to estimate pairwise similarity of the samples by Dice coefficient *D*_c_ = 2*j*/(*a* + *b*), where *j* is the number of bands common to both samples, *a* is the number of bands in sample A, and *b* is the number of bands in sample B. Obtained data were analyzed and clustered by unweighted pair group method using arithmetic average (UPMGA) algorithm. This allows comparison between fingerprints using solely the presence or absence of fluorescence bands/peaks and without taking into account its intensity.

The structural diversity of the microbial community was examined by the Shannon index of general diversity *H*′ [16]. *H*′ was calculated on the basis of bands on the gel tracks using densitometric curves. The intensity of the bands was reflected as peak heights in the densitometric curve. The equation for the Shannon index is: $H^{\prime } \, = \, - \Upsigma \left( {n_{i} /N} \right){ \log }\left( {n_{i} /N} \right),$ where *n*_*i*_ is the height of the peak and *N* the sum of all peak heights of the densitometric curve.

The Pearson correlation analysis was used to establish the relationship between biogas/methane production and relative abundance of MT1, MT2 bands/peaks. The Pearson coefficient (*P*c) ranges between −1 and +1, where (*P*c) = −1 or +1 means the perfect correlation while 0 means an absence of a relationship. Correlations are considered statistically significant at the 95 % confidence interval (*P* < 0.05). The Pearson’s product correlation coefficient was analyzed using Statistica 10.0 (StatSoft, USA).

### DNA Cloning and Sequencing

Dominant bands visible in polyacrylamide gel were excised, transferred into 50 μL sterile water and frozen in −20 °C for 24 h. The samples were thawed at room temperature and gel fragments were homogenized. Eluted PCR products were then reamplified using the same primers set and purified with Clean-up kit (A&A Biotechnology, Gdynia, Poland).

Sequencing reactions were carried out with ABI 3730XL (Applied Biosystems, USA) performed by Macrogen Europe (Amsterdam, Netherlands). All reactions were run following the manufacturer’s protocols. Obtained sequences were aligned using ClustalW program [18], and analyzed with the BLASTN algorithm [1]. The nucleotide sequences were deposited in GenBank under accession numbers JX989819–JX989820.

## Results

The designed PCR primer pair allowed amplification of Ribosomal Intergenic Spacer plus 202 bp of *16S* and 170 bp of *23S rRNA* genes. Since the length of DNA fragments corresponding to both genes suggests that only amplicons longer than 372 bp could be identified as RISs, only amplicons in the range between 400 and 1,000 bp were analyzed in this study.

**Fig. 2:**
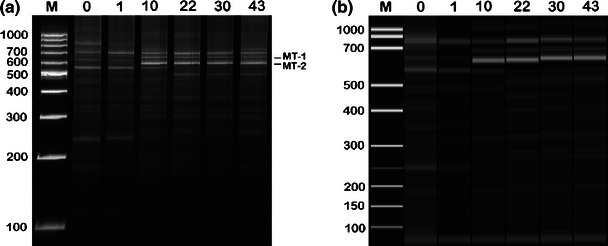
The banding profiles of the archaeal community obtained by the resolving of the ribosomal intergenic spacer region in polyacrylamide gel (**a**) and the application of automated microfluidics-based (MF-ARISA) (**b**). The numbers above the lanes show the sampling day, *0* inoculum, *M* molecular weight marker. Bands marked as MT-1 and MT-2 were excised and sequenced

**Fig. 3:**
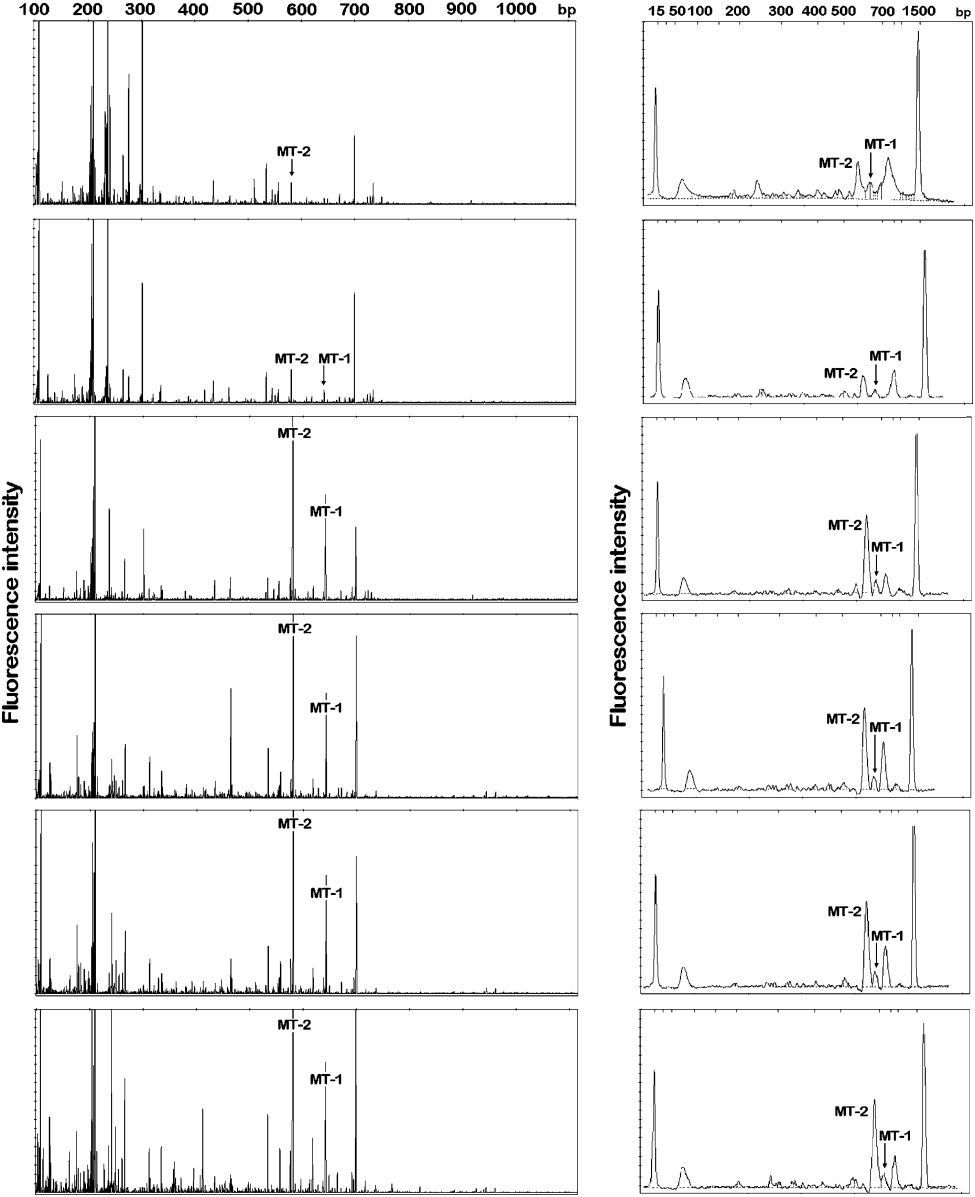
Electrophoregrams presenting the archaeal community obtained using *GA-ARISA* and *MF-ARISA*. Marked peaks (*MT-1* and *MT-2*) represent dominant bands excised from the gel and sequenced. The specific size markers (15 and 1,500 bp) that were added to each MF-ARISA are denoted by *asterisks*

The changes in methanogenic *Archaea* consortia have been experimentally studied by RISA, MF-ARISA, and GA-ARISA. All applied approaches reflected changes in the structure of analyzed *Archaea* derived from an anaerobic digester and utilized for biogas synthesis. The results of RISA and MF-ARISA, in the form of gel electrophoresis, are shown on Fig. 2, whereas Fig. 3 presents the outcomes of MF- and GA-ARISA as fluorograms.

**Fig. 4:**
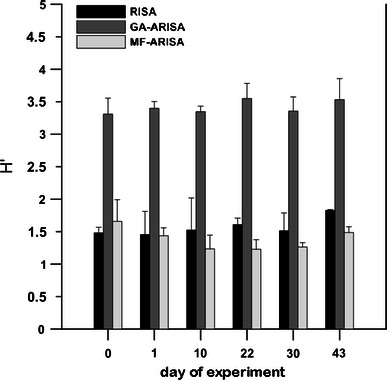
Shannon diversity index (*H′*) estimated for all applied approaches: *RISA—*standard analysis performed by polyacrylamide gel electrophoresis, capillary electrophoresis (*GA-ARISA*), and microfluidics-based (*MF-ARISA*). Values are means from three replicates given with standard errors

The total band/peak numbers differ depending on the method and analyzed sample. The band/peak number was the highest for GA-ARISA, intermediate for MF-ARISA and lowest for RISA. In the case of RISA and MF-ARISA, the highest number of dominant Ribosomal Intergenic Spacers fragments was in the range between 500 and 800 bp. However, for GA-ARISA, many peaks in the range between 200 and 300 bp were observed. A comparison of the total archaeal diversity obtained using all techniques is shown in Fig. 4. The values of means resulted from triplication with standard deviation. The richness values, measured using the Shannon equation, were lowest for MF-ARISA (from 1.22 ± 0.15 to 1.65 ± 0.33) and highest in GA-ARISA (from 3.3 ± 0.24 to 3.54 ± 0.23). The intermediate measures were denoted for RISA, which were in the range from 1.48 ± 0.08 to 1.82 ± 0.01.

**Fig. 5:**
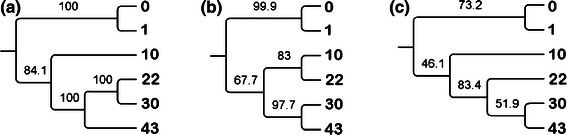
UPGMA trees representing genetic similarity of the archaeal community profiles characterized by three variants of RISA: **a** standard analysis performed by polyacrylamide gel electrophoresis, **b** microfluidics-based variant (MF-ARISA) and that conducted by capillary electrophoresis (GA-ARISA). The numbers indicate the sampling day, *0* inoculum

Successive changes in archaeal communities were observed during the start-up period of anaerobic digestion. Many DNA bands were present in both the seeds and samples taken after 1 day of digestion. On the 10th day of the process, a shift in microbial community structure was observed. By this time, the DNA fingerprint was clearly different from that observed during the start-up. After this time, changes in microbial communities were insignificant and a predominance of bands in the range 600 and 700 bp was observed. The same tendency was ascertained in the case of all applied methods. To analyze the direction of the archaeal community changes during anaerobic digestion, the genetic distance between samples was calculated using the Dice coefficient method. The obtained results were clustered by the UPMGA algorithm and are shown on Fig. 5. Clustering analysis showed two main groups, the first comprised of inoculum and a sample taken 24 h later, while the second cluster joined the rest of the samples. The second group was diversified depending on the utilized approach. The highest values of bootstrap were estimated for the tree obtained by RISA application, whereas the lowest for the tree constructed using GA-ARISA (Fig. 5).

To determine the dominant micro-organisms, two DNA bands were excised from the gel and were re-amplified, cloned, and sequenced. The obtained DNA sequences were analyzed primarily by comparison with corresponding sequences deposited in GenBank. The performed analysis revealed that both sequences (described as MT-1 and MT-2) were phylogenetically related to *Methanosarcina barkeri* (CP000099). The DNA sequence of the MT-1 band showed 98 % (and the MT-2 showed 99 %) similarity to the above-mentioned DNA sequence of *M. barkeri*. The analyzed DNA sequences were different in length. The MT-1 DNA sequence was 652 bp long, whereas MT-2 was only 590 bp. Taking into consideration the fact that amplicons possess fragments belonging to *16S* and *23S* *rRNA* genes, the lengths of RIS were calculated as 280 bp (MT-1) and 218 bp (MT-2). The observed difference was revealed only from the absence of tRNA^Ala^ in the MT-2 sequence. Both amplicons were recognized as the peaks in GA-ARISA and MF-ARISA fluorograms (Fig 3). The correlation between biogas/methane production, and relative abundance of MT1, MT2 bands/peaks have been performed using Pearson correlation analysis. With the exception of the MF-ARISA MT1 peak, all peaks detected were strongly correlated with biogas and methane volumes. The lowest statistically significant values of the Pearson coefficient were noticed for MT2 peak obtained also by MF-ARISA (Table 4).

**Table 4:** Pearson’s correlation between values of biogas/methane production and relative abundance of MT1 and MT2 bands/peaks obtained by each of applied methods

Parameter	RISA		MF-ARISA		GA-ARISA	
	MT1	MT2	MT1	MT2	MT1	MT2
Biogas	0.94 *P* = 0.015	0.99 *P* = 0.001	0.66	0.91 *P* = 0.03	0.96 *P* = 0.011	0.94 *P* = 0.018
Methane	0.94 *P* = 0.018	0.98 *P* = 0.002	0.66	0.90 *P* = 0.03	0.96 *P* = 0.08	0.95 *P* = 0.015

Significant coefficients values are accompanied with probability values

## Discussion

To guarantee the correct design and application of anaerobic treatment systems, knowledge of the technological aspects, biochemistry and microbiology of anaerobic digestion is essential [6] However, the terminal phase of anaerobic treatment-methanogenesis, is still not completely understood, especially in systems where complex substrates are utilized for biogas production. A wide variety of molecular methods have been developed to assess the role of archaeal methanogens in such processes. Traditional examination of the microbial ecology in the environment is based on the microbes’ cultivation under laboratory conditions, although for many micro-organisms, especially *Archaea*, culture-dependent studies can be difficult [11]. However, since molecular analysis is free of limitations that are typical for culture-dependent methods, such an approach is preferred for a methodology for the examination of methane-producing micro-organisms. Unfortunately, many of these DNA-based methods often provide results which are ambiguous and difficult to interpret. In addition, they usually cannot be automated and are restricted by the limited number of samples that can be analyzed in one run. Thus, our intention was to examine the usefulness of RISA-a method that could be free of this limitation.

In this study, three variants of RISA were compared to find the best solutions for controlling changes in methanogenic archaeal consortia during anaerobic treatment of silage. The standard method that relies on amplicon resolution on polyacrylamide gel was matched against the automated methods performed by an Applied Biosystems 3130 Genetic Analyzer (GA-ARISA) and microfluidics-based Agilent 2100 Bioanalyzer (MF-ARISA).

All applied techniques showed similar clustering for the samples taken from the digester over time. Although, the highest values of bootstrap were estimated for the tree obtained by RISA application (Fig. 5a), the most reliable picture of archaeal community changes was gained using GA-ARISA (Fig. 5c), where the direction of genetic similarity changes was concordant with the time of sampling. The lower bootstrap support values are mainly due to the fact that this approach led to detect the highest number of taxons, that was supported by Shannon index values (Fig. 4). An additional advantage of GA-ARISA is its suitability for the routine analysis of a large number of samples (which, in the case of the Applied Biosystems 3130 Genetic Analyzer could be up to 96). For MF-ARISA, the maximum number of samples analyzed in a single run is 12 which, together with low resolution power, are the main disadvantages of this approach. The inability to load many samples on the same gel and the potential of gel-to-gel variation is a major drawback of gel-based RISA. However, the advantage of RISA is that it can be coupled with additional approaches to identify differentiating bands. Excision of selected bands and its detailed analysis by sequencing can lead to the phylogenetic affiliation of micro-organisms that are represented in the fingerprint. However, along with development of annotated 16S‐RIS‐23S region collections, the information on their source organisms could enrich ARISA by allowing micro-organism identification based on RISs lengths.

The two dominant bands in polyacrylamide gel (MT-1 and MT-2) were excised and characterized by DNA sequencing. The obtained results revealed that both analyzed sequences are most similar to the sequence of *Methanosarcina barkeri.* Although the lengths of analyzed sequences were different than those released from the absence of tRNA^Ala^ in the MT-2 sequence, the rest of this region was identical. Because both bands appeared at the same time (the 10th day of the process) and the intensities of these bands were similar until the end of anaerobic digestion, it is highly probable that both analyzed intergenic spacer regions originated from one micro-organism. The presence of two, different ribosomal operons was previously proven in other archaeon *Haloarcula marismortui* [3]. In this species, one of the operons possessed RIS lacking genes coding for tRNA^Ala^ and tRNA^Cys^. This same phenomena was observed in *Enterococcus hirae* ATCC 9790 [15].

The presence of *Methanosarcina* sp. is typical for digesters with high levels of N–NH_4_^+^and VFAs [4]. According to Raskin et al. [13], *Methanosarcina* sp. were the most abundant methanogenic archaea in samples taken from acetate-fed laboratory chemostats. In our experiment, bands corresponding to *Methanosarcina barkeri* appeared on the 10th day of experiment when total biogas and methane production increased rapidly. It suggests that *Methanosarcina barkeri* was the main biogas producer in the performed experiment. The development of this species was promoted by high levels of N–NH_4_^+^ and VFAs (Table 2). The Pearson correlation analysis showed that there is correlation between biogas/methane production and relative abundance of MT1, MT2 bands/peaks, that can support the finding that *M. barkeri* is responsible for biogas production in the studied digester.

In conclusion, our results indicate that all methods are useful as fingerprinting techniques for assessing archaeal species changes and diversity in anaerobic environments. However, the results of this study indicate that ARISA performed by a genetic analyzer (GA-ARISA) is more accurate and reproducible than other tested approaches. Although, its performance requires specialized equipment, experienced staff and its execution time is quite long, the reliable results compensate for these disadvantages. Therefore, it could be concluded that the designed PCR primer pair, combined with the application of capillary electrophoresis, could be a powerful technique for methanogenic *Archaea* analysis.

## References

[1] Altschul SF, Madden TL, Schäffer AA, Zhang J, Zhang Z, Miller W, Lipman DJ (1997). Gapped BLAST and PSI-BLAST: a new generation of protein database search programs. Nucleic Acids Res.

[2] American Public Health Association (APHA) (1992). Standard methods for the examination of water and wastewater.

[3] Dennis PP, Ziesche S, Mylvaganam S (1998). Transcription analysis of two disparate rRNA operons in the halophilic archaeon *Haloarcula marismortu*i. J Bacteriol.

[4] Demirel B, Scherer P (2008). The roles of acetotrophic and hydrogenotrophic methanogens during anaerobic conversion of biomass to methane: a review. Rev Environ Sci Biotechnol.

[5] Fisher MM, Triplett EW (1999). Automated approach for ribosomal intergenic spacer analysis of microbial diversity and its application to freshwater bacterial communities. Appl Environ Microbiol.

[6] Lema JM, Omil F (2001). Anaerobic treatment: a key technology for a sustainable management of wastes in Europe. Water Sci Technol.

[7] Liu Y, Whitman WB (2008). Metabolic, phylogenetic, and ecological diversity of the methanogenic archaea. Ann NY Acad Sci.

[8] McMahon KD, Zheng D, Stams AJM, Mackie RI, Raskin L (2004). Microbial population dynamics during start-up and overload conditions of anaerobic digesters treating municipal solid waste and sewage sludge. Biotechnol Bioeng.

[9] Morris R (2009) Relating methanogen community structure to function in anaerobic wastewater digester. Dissertation, Marquette University

[10] Muyzer G, de Waal EC, Uitterlinden AG (1993). Profiling of complex microbial populations by denaturing gradient gel electrophoresis analysis of polymerase chain reaction-amplified genes encoding for 16S rRNA. Appl Environ Microbiol.

[11] Nelson MC (2011) An integrated investigation of the microbial communities underpinning biogas production in anaerobic digestion systems. Dissertation, The Ohio State University

[12] Ranjard L, Poly F, Lata JC, Mougel C, Thioulouse J, Nazaret S (2001). Characterization of bacterial and fungal soil communities by automated ribosomal intergenic spacer analysis fingerprints: biological and methodological variability. Appl Environ Microbiol.

[13] Raskin L, Poulsen LK, Noguera DR, Rittmann BE, Stahl DA (1994). Quantification of methanogenic groups in anaerobic biological reactors by oligonucleotide probe hybridization. Appl Environ Microbiol.

[14] Rozzi A, DiPinto AC (1994). Start-up and automation of anaerobic digesters with automatic bicarbonate control. Bioresour Technol.

[15] Sechi LA, Daneo-Moore L (1993). Characterization of intergenic spacers in two rrn operons of *Enterococcus hirae* ATCC 9790. J Bacteriol.

[16] Shannon CE, Weaver W (1963). The mathematical theory of communication.

[17] Speece RE, Boonyakitsombut S, Kim M, Azbar N, Ursillo P (2006). Overview of anaerobic treatment: thermophilic and propionate implications. Water Environ Res.

[18] Thompson JD, Higgins DG, Gibson TJ (1994). CLUSTAL W: improving the sensitivity of progressive multiple sequence alignment through sequence weighting, positions- specific gap penalties and weight matrix choice. Nucleic Acids Res.

[19] White D (2000). The physiology and biochemistry of prokaryotes.

[20] Yu Y, Kim J, Hwang S (2006). Use of real-time PCR for group-specific quantification of aceticlastic methanogens in anaerobic process: population dynamics and community structures. Biotechnol Bioeng.

